# Supplementary material for: Phi-Value and NMR Structural Analysis of a Coupled Native-State Prolyl Isomerization and Conformational Protein Folding Process
Source: Biomolecules. 2025 Feb 10;15(2):259. doi: 10.3390/biom15020259 (PMC11852654; doi:10.3390/biom15020259)
Supplement: Supplementary file 1 [file biomolecules-15-00259-s001.zip › biomolecules-3446412-supplementary.pdf]

Phi-Value and NMR Structural Analysis of a Coupled Native-State Prolyl Isomerization and Conformational Protein Folding Process

Ulrich Weininger [1], Maximilian von Delbrück [2], Franz X. Schmid [2] and Roman P. Jakob [3,*]

[1] Institute of Physics, Biophysics, Martin-Luther-University Halle-Wittenberg, 06120 Halle (Saale), Germany; ulrich.weininger@physik.uni-halle.de
[2] Laboratorium für Biochemie und Bayreuther Zentrum für Molekulare Biowissenschaften, Universität Bayreuth, 95447 Bayreuth, Germany; delbrueck@knauer.net (M.v.D.); fx.schmid@uni-bayreuth.de (F.X.S.)
[3] Focal Area Structural Biology, Biozentrum, University of Basel, Spitalstrasse 41, 4056 Basel, Switzerland
* Correspondence: roman.jakob@unibas.ch; Tel.: +41-61-267-2103; Fax: +41-61-267-2109

Supplementary Materials

Page 2, Supplementary Table S1: Stability data for N2′ and the variants
Page 4, Supplementary Table S2: Unfolding and refolding kinetics of N2′ variants
Page 6, Supplementary Table S3: Statistics of the NMR structure determination

Page 7, Supplementary Figure S1: Functional characterization and stability of the N2′ variants.
Page 8, Supplementary Figure S2: Thermal induced unfolding transitions of N2' variants.
Page 9, Supplementary Figure S3: Urea induced equilibrium unfolding transitions.
Page 10, Supplementary Figure S4: Folding kinetics.
Page 11, Supplementary Figure S5: Chevron plots.
Page 12, Supplementary Figure S6: NMR structural analysis.
Page 12, Supplementary References

Supplementary Table S1: Stability data for N2′ and the variants

variant	T_M (°C)	$\Delta G_D^{32°C}$ (kJ mol^{-1})	$\Delta G_D^{15°C}$ (kJ mol^{-1})	ΔH_D (kJ mol^{-1})	[Urea]$_M$ (M)	$\Delta G_D^{15°C}$ (0 M) (kJ mol^{-1})	$\Delta G_D^{15°C}$ (2 M) (kJ mol^{-1})	$\Delta\Delta G_D^{15°C}$ (0 M) (kJ mol^{-1})	$\Delta\Delta G_D^{15°C}$ (2 M) (kJ mol^{-1})	*m*-value (kJ mol^{-1} M^{-1})
	Thermal induced unfolding				Urea induced unfolding					
N2'	38.4	6.2	18.7	320.2	2.7	17.1	4.7			-6.7
I103A	28.4	-3.8	11.5	300.7	1.5	11.9	-4.0	-5.2	-8.6	-7.9
L106A	24.6	-7.8	8.3	288.4	1.0	7.5	-8.3	-9.6	-13.0	-7.9
Y110A	29.3	-2.5	11.0	275.5	1.5	10.0	-3.4	-7.1	-8.0	-6.7
P112A	34.3	2.2	15.4	304.8	2.2	14.0	1.3	-3.1	-3.4	-6.3
T114A	28.5	-3.6	11.5	293.1	1.4	9.1	-3.9	-8.0	-8.6	-6.5
Q116A	37.3	5.0	17.3	308.4	2.7	16.9	4.3	-0.2	-0.4	-6.3
P118A	37.6	5.2	17.3	306.8	2.7	16.5	4.3	-0.6	-0.4	-6.2
P121A	33.8	1.7	14.8	299.7	2.2	14.2	1.3	-2.9	-3.4	-6.5
P123A	35.6	3.5	16.8	314.7	2.4	16.5	2.7	-0.6	-2.0	-6.8
N132A	34.7	2.7	16.0	310.8	2.2	13.0	1.2	-4.1	-3.5	-6.0
R142A	42.4	9.6	20.6	322.2	3.5	20.0	8.6	2.9	3.9	-5.7
Q145A	39.1	6.8	19.6	321.5	2.8	18.3	5.4	1.2	0.7	-6.5
G146A	28.5	-3.4	11.1	288.5	1.4	9.2	-3.8	-7.9	-8.5	-6.4
V150A	24.4	-7.7	8.4	281.8				-9.1*		
Y151A	24.2	-8.3	8.1	290.0				-9.7*		
G153A	29.4	-2.7	12.5	306.1	1.4	9.0	-4.0	-8.1	-8.7	-6.6
V155A	31.2	-0.8	13.9	310.1	1.7	13.3	-2.2	-3.8	-6.8	-7.7
Y166A	26.4	-6.0	10.2	303.9	1.1	9.0	-7.8	-8.1	-12.5	-8.4
T169A	35.5	3.5	16.9	317.7	2.3	16.4	1.9	-0.7	-2.8	-7.2
M176A	26.1	-5.7	8.8	270.9	0.9	6.5	-6.5	-10.6	-11.2	-5.9
D178A	37.7	5.6	18.4	321.9	2.5	15.2	3.0	-1.9	-1.7	-6.1
W181A	34.3	2.1	13.9	280.0	2.0	13.5	0.0	-3.6	-4.7	-6.7
F185A	28.6	-3.2	10.3	270.2	1.2	7.9	-5.5	-9.2	-10.2	-6.9
D187A	29.7	-2.3	12.0	292.4	1.4	9.9	-4.2	-7.2	-8.9	-7.0
F190A	33.6	1.4	13.6	280.6	1.8	12.3	-1.4	-4.8	-6.1	-6.8
H191A	35.2	2.9	14.7	286.7	2.0	12.8	0.0	-4.3	-4.7	-6.4
N195A	36.7	4.5	17.3	313.3	2.5	17.3	3.5	0.2	-1.2	-6.9
L198P	47.3	14.2	24.5	343.7	4.3	24.9	13.3	7.8	8.7	-5.8
V200A	34.2	2.1	15.1	299.8	1.8	12.9	-1.4	-4.2	-6.1	-7.1
Y203A	31.1	-0.8	11.8	271.8	1.4	10.2	-4.3	-6.9	-9.0	-7.2

The Supplementary Table S1 presents stability parameters for the N2' and variants, determined using a two-state model from thermal (columns 2 to 5) and urea-induced unfolding transitions (columns 6 to 12). For each protein, the T_M values, the free enthalpy of unfolding ΔG_D, at 15 °C and 32 °C, the Van't Hoff enthalpy of unfolding, ΔH_D, the Urea concentration at the transition midpoint, $[\text{Urea}]_M$, the free enthalpy of unfolding $\Delta G_D^{15\,°C}$ at 0 M and 2 M urea and 15°C, the difference in free energy of unfolding at 15 °C between the N2' variant and the wild-type protein at 0 and 2 M urea, $\Delta\Delta G_D^{15\,°C}$ (0 M) and $\Delta\Delta G_D^{15\,°C}$ (2 M), and the *m*-value are given. Free energy changes marked with an asterisk (*) were extrapolated to 15 °C from thermal transition data due to insufficient protein yield for urea-induced equilibrium measurements. Thermal induced denaturation was monitored at 222 nm using circular dichroism (CD) spectroscopy in a quartz cuvette with a 1 cm path length. Measurements were conducted at 100 mM potassium phosphate buffer, pH 7.0, with a heating rate of 60 K/h and a bandwidth of 1.0 nm. Data points were recorded every 0.2 K. Urea induced transitions were characterized by fluorescence changes at 340 nm (except for N2'-W181A, measured at 305 nm) upon excitation at 280 nm at 15 °C, using protein concentrations of 1.0 μM in 100 mM potassium phosphate buffer, pH 7.0, with excitation and emission bandwidths of 3 nm and 5 nm, respectively. The wild-type N2' data are taken from Table 1 of Jakob & Schmid, 2008 [1].

Supplementary Table S2. Unfolding and refolding kinetics of variants of N2′

variant	k_{NU} (2 M)	k_{UN} (2 M)	m_{NU}	m_{UN}	m	[Urea]$_M$	ΔG_D (2 M)	β_T	$\Delta\Delta G_D$ (2 M)	$\Delta\Delta G_{UN}$‡(2 M)	$\Delta\Delta G_{NU}$‡(2 M)	Φ-value (2 M)	
	(s^{-1})	(s^{-1})	(kJ mol^{-1} M^{-1})	(kJ mol^{-1} M^{-1})	(kJ mol^{-1} M^{-1})	(M)	(kJ mol^{-1})		(kJ mol^{-1})	(kJ mol^{-1})	(kJ mol^{-1})	Φ_{trans}	Φ_{cis}
wild-type													
cis	8.72	0.15	-1.42	0.79	5.30	3.80	9.80	0.64	0.00	0.00	0.00		
trans	0.13	0.19	-1.71	0.77	6.00	1.80	-0.93	0.69	0.00	0.00	0.00		
I103A									-8.6				
cis	3.96	2.65	-1.42	0.79	5.29	2.18	0.96	0.64		-1.89	-6.95	0.2	0.2
trans		2.78		0.78							-6.48		0.3
L106A									-13.0				
cis	5.24	14.32	-1.40	0.78	5.2	1.50	-2.41	0.64		-1.22	-10.99	0.1	0.2
trans		14.47		0.78							-10.42		0.2
Y110A									-8.0				
cis	4.61	6.38	-1.40	0.82	5.31	1.85	-0.78	0.63		-1.52	-9.05	0.2	-0.1
trans		7.94		0.77							-8.98		-0.1
P112A									-3.4				
cis	10.34	0.51	-1.42	0.86	5.46	3.32	7.23	0.62		0.41	-2.99	-0.1	0.1
trans	0.14	0.54	-1.72	0.85	6.14	1.47	-3.25	0.67		0.22	-2.55	-0.1	0.3
T114A									-8.6				
cis	4.43	7.51	-1.48	0.81	5.49	1.75	-1.26	0.65		-1.62	-9.45	0.2	-0.1
trans	0.13	4.85	-1.73	0.77	5.99	0.29	-8.61	0.69		0.12	-7.81	0.0	0.1
Q116A													
cis	9.12	0.67	-1.41	0.78	5.25	3.19	6.24	0.64					
trans	0.13	0.71	-1.73	0.77	5.99	1.33	-4.02	0.69					
P118A													
cis	9.44	0.20	-1.40	0.82	5.30	3.74	9.25	0.63					
trans	0.13	0.18	-1.72	0.86	6.17	1.88	-0.75	0.67					
P121A									-3.4				
cis	10.08	1.29	-1.41	0.81	5.33	2.93	4.93	0.63		0.35	-5.22	-0.1	-0.5
trans	0.13	1.07	-1.71	0.86	6.16	1.19	-5.01	0.67		0.11	-4.19	0.0	-0.2
P123A									-2.0				
cis	10.19	0.39	-1.42	0.80	5.32	3.47	7.84	0.64		0.37	-2.34	-0.2	-0.2
trans	0.14	0.41	-1.73	0.79	6.04	1.57	-2.59	0.69		0.23	-1.90	-0.1	0.0
N132A									-3.5				
cis	4.41	0.35	-1.42	0.80	5.32	3.13	6.04	0.64		-1.63	-2.13	0.5	0.4
trans	0.08	0.41	-1.73	0.78	6.01	1.33	-4.03	0.69		-1.21	-1.88	0.3	0.5
R142A									3.9				
cis	10.69	0.32	-1.42	0.74	5.18	3.62	8.41	0.66		0.49	-1.89	0.1	0.5
trans	0.48	0.33	-1.72	0.73	5.88	2.15	0.85	0.70		3.18	-1.40	0.8	0.4
Q145A													
cis	16.87	0.13	-1.43	0.90	5.57	4.09	11.62	0.61					
trans	0.19	0.21	-1.71	0.78	5.96	1.95	-0.32	0.69					
G146A									-8.5				
cis	0.45	0.36	-1.42	0.93	5.63	2.09	0.52	0.61		-7.12	-2.16	0.8	0.7
trans		0.36		0.92							-1.60		0.8
V150A									-13.9				
cis	3.73	2.25	-1.41	0.78	5.25	2.23	1.22	0.65		-2.03	-6.55	0.2	0.5
trans		2.29		0.77							-6.01		0.6
Y151A									-14.5				
cis		3.08	-1.42	0.78				0.65			-7.31		0.5
trans		2.91		0.77							-6.58		0.5
G153A									-8.7				
cis	0.67	0.35	-1.41	0.79	5.28	2.29	1.52	0.64		-6.15	-2.13	0.7	0.8
trans		0.37		0.78							-1.65		0.8
V155A									-6.8				
cis	2.88	0.35	-1.42	0.80	5.32	2.94	5.02	0.64		-2.65	-2.13	0.4	0.7
trans	0.06	0.35	-1.72	0.81	6.05	1.32	-4.10	0.68		-1.67	-1.51	0.2	0.8

continued on the next page

variant	k_{NU} (2 M) (s^{-1})	k_{UN} (2 M) (s^{-1})	m_{NU} (kJ mol^{-1} M^{-1})	m_{UN} (kJ mol^{-1} M^{-1})	m (kJ mol^{-1} M^{-1})	[Urea]$_M$ (M)	ΔG_D (2 M) (kJ mol^{-1})	β_T	$\Delta\Delta G_D$ (2 M) (kJ mol^{-1})	$\Delta\Delta G_{UN}$‡(2 M) (kJ mol^{-1})	$\Delta\Delta G_{NU}$‡(2 M) (kJ mol^{-1})	Φ-value (2 M) Φ_{trans}	Φ_{cis}
wild-type													
cis	8.72	0.15	-1.42	0.79	5.30	3.80	9.80	0.64	0.00	0.00	0.00		
trans	0.13	0.19	-1.71	0.77	6.00	1.80	-0.93	0.69	0.00	0.00	0.00		
Y166A									-12.5				
cis	1.85	1.71	-1.44	0.77	5.29	2.03	0.19	0.65		-3.72	-5.90	0.3	0.5
trans	0.05	1.75	-1.71	0.78	5.96	0.60	-8.33	0.69		-2.04	-5.36	0.2	0.6
T169A									-2.8				
cis	10.28	0.37	-1.42	0.87	5.51	3.44	7.93	0.62		0.40	-2.27	-0.1	0.2
trans	0.13	0.39	-1.72	0.87	6.21	1.57	-2.65	0.66		0.06	-1.78	0.0	0.4
M176A									-11.2				
cis	1.61	6.79	-1.43	0.72	5.16	1.33	-3.45	0.66		-4.05	-9.20	0.4	0.2
trans	0.00	6.96		0.71							-8.67		0.2
D178A													
cis	8.70	0.22	-1.41	0.78	5.25	3.68	8.84	0.64					
trans	0.12	0.23	-1.71	0.78	5.95	1.74	-1.58	0.69					
W181A									-4.7				
cis	8.13	0.89	-1.52	0.93	5.85	2.91	5.31	0.62		-0.17	-4.33	0.0	0.1
trans	0.11	1.02	-1.73	0.90	6.30	1.16	-5.27	0.66		-0.27	-4.07	0.1	0.1
F185A									-10.2				
cis	5.39	6.07	-1.42	0.55	4.71	1.94	-0.28	0.72		-1.15	-8.94	0.1	0.1
trans	0.00	6.71		0.53							-8.58		0.2
D187A									-8.9				
cis	4.34	5.34	-1.43	0.56	4.76	1.90	-0.49	0.72		-1.67	-8.63	0.2	0.0
trans	0.00	5.49		0.55							-8.10		0.1
F190A									-6.1				
cis	11.61	1.69	-1.43	0.80	5.33	2.87	4.62	0.64		0.69	-5.87	-0.1	0.0
trans	0.12	2.18	-1.71	0.73	5.84	0.80	-7.01	0.70		-0.19	-5.89	0.0	0.0
H191A									-4.7				
cis	5.35	1.12	-1.44	0.70	5.13	2.73	3.75	0.67		-1.17	-4.88	0.2	0.0
trans	0.13	1.00	-1.71	0.73	5.84	1.17	-4.87	0.70		0.07	-4.02	0.0	0.1
N195A													
cis	8.14	0.23	-1.43	0.90	5.59	3.53	8.54	0.61					
trans	0.15	0.25	-1.71	0.90	6.24	1.80	-1.23	0.66					
L198P									8.7				
cis	6.91	0.00	-1.43	0.81	5.36	5.50	18.97	0.64		-0.56	9.72	-0.1	-0.1
trans	0.17	0.00	-1.72	0.78	6.00	3.56	9.42	0.69		0.70	9.65	0.1	-0.1
V200A									-6.1				
cis	9.10	2.04	-1.38	0.72	5.03	2.71	3.58	0.66		0.10	-6.33	0.0	0.0
trans	0.11	2.03	-1.73	0.74	5.93	0.82	-6.98	0.70		-0.32	-5.72	0.1	0.1
Y203A									-9.0				
cis	6.82	4.25	-1.39	0.59	4.75	2.24	1.13	0.70		-0.59	-8.08	0.1	0.1
trans	0.09	5.12	-1.69	0.52	5.30	0.19	-9.61	0.76		-0.75	-7.93	0.1	0.1

The kinetic parameters were determined in 100 mM K phosphate (pH 7.0) at 15 °C, pH 7.0, from the kinetic chevrons shown in Supplementary Figure 3 after a linear two-state analysis as described in Materials and Methods. The microscopic rate constants of refolding k_{UN} (s^{-1}) and unfolding k_{NU} (s^{-1}) at 2 M urea are given. m_{UN} (kJ mol^{-1} M^{-1}) and m_{NU} (kJ mol^{-1} M^{-1}) are the kinetic m values for refolding and unfolding, respectively. β_T is the Tanford value. ΔG_D is the Gibbs free energy of denaturation calculated from the chevron plots. $\Delta\Delta G_{UN}$‡ (2M) is the difference in activation free energy of refolding between the variants and the reference protein (N2′) in the presence of 2 M urea. $\Delta\Delta G_{NU}$‡ (2M) is the difference in Gibbs free energy of unfolding between the variants and the reference protein (N2′) in the presence of 2 M urea. All ΔG and $\Delta\Delta G$ are given in kilojoules per mole. Φ_{trans} and Φ_{cis} are the calculated Φ values in the presence fo 2 M urea. Φ values are not given when $\Delta\Delta G_D$ is smaller than 2 kJ mol^{-1}.

Supplementary Table S3. Statistics of the NMR structure determination

NMR distance and dihedral constraints	***cis* form**	***trans* form**
Total NOE [a]	2877	2864
Intraresidue [a]	1094	1089
sequential ($\|i-j\|$ =1) [a]	609	609
medium ($\|i-j\|$ <4) [a]	313	307
long ($\|i-j\|$ >5) [a]	861	859
Total dihedral angle restraints	140	140
ϕ	70	70
ψ	70	70
RDC	34	34
H-bonds	24	24
Structure statistics		
Violations (mean and SD)		
NOE constraints, Å	0.03±0.02	0.03±0.02
Dihedral angle constraints, °	1.3±0.2	2.9±0.2
H-bonds, Å	0.03±0.02	0.11±0.04
Ramachandran analysis		
Most favored, %	89.9	89.7
Additionally allowed, %	9.2	9.7
Generously allowed, %	0.6	0.5
Disallowed, %	0.3	0.1
Average pairwise rmsd, Å		
Heavy, all	2.6±0.5	2.0±0.6
Backbone, all	2.0±0.5	1.6±0.6
Heavy, 2nd structure	0.82±0.07	0.83±0.11
Backbone, 2nd structure	0.38±0.06	0.45±0.08

[a] derived from the automated assignment of ambiguous NOEs

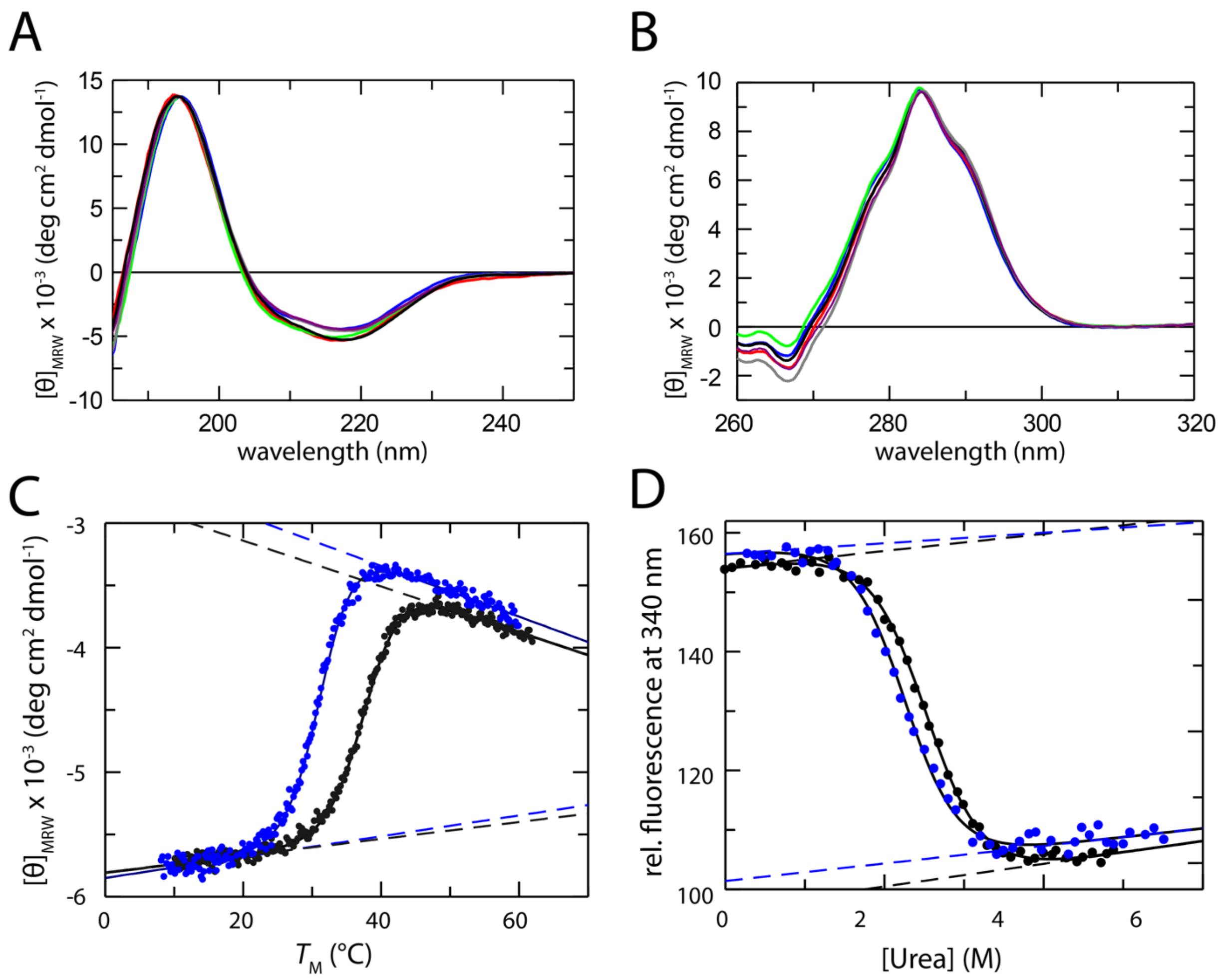

Supplementary Figure S1. Functional characterization and stability of the N2′ variants. The far-UV spectra (**A**) and near-UV spectra (**B**) of (–) N2′; (–) N2′-P112A; (–) N2′-N132A; (–) N2′-M176A; (–) N2′-D187A; (–) N2′-V200A. The far-UV-CD spectra of 5μM protein were recorded from 185 to 250 nm at 15 °C in a 1 mm path-length cuvette in 10 mM potassium phosphate buffer, pH 7.0. The near-UV CD spectra were recorded from 260 to 320 nm at 15 °C in a 10 mm path-length cuvette with 50 μM in 100 mM potassium phosphate buffer, pH 7.0. Measurement parameters: bandwidth 2 nm; resolution 0.2 s; scan speed 100 nm/min. The spectra were measured ten times and averaged. (**C**) Thermal induced unfolding for the N2′ variants Q116A (black) and V155A (blue). Plotted is the molar ellipticity $[\theta]_{MRW}$ at 222 nm as a function of the temperature. (**C**) Urea induced equilibrium transitions of the N2' variants T169A (blue) and N195A (black). The solid lines show the fits to a two-state model, the broken lines correspond to the baselines of the native and denatured N2' variants.

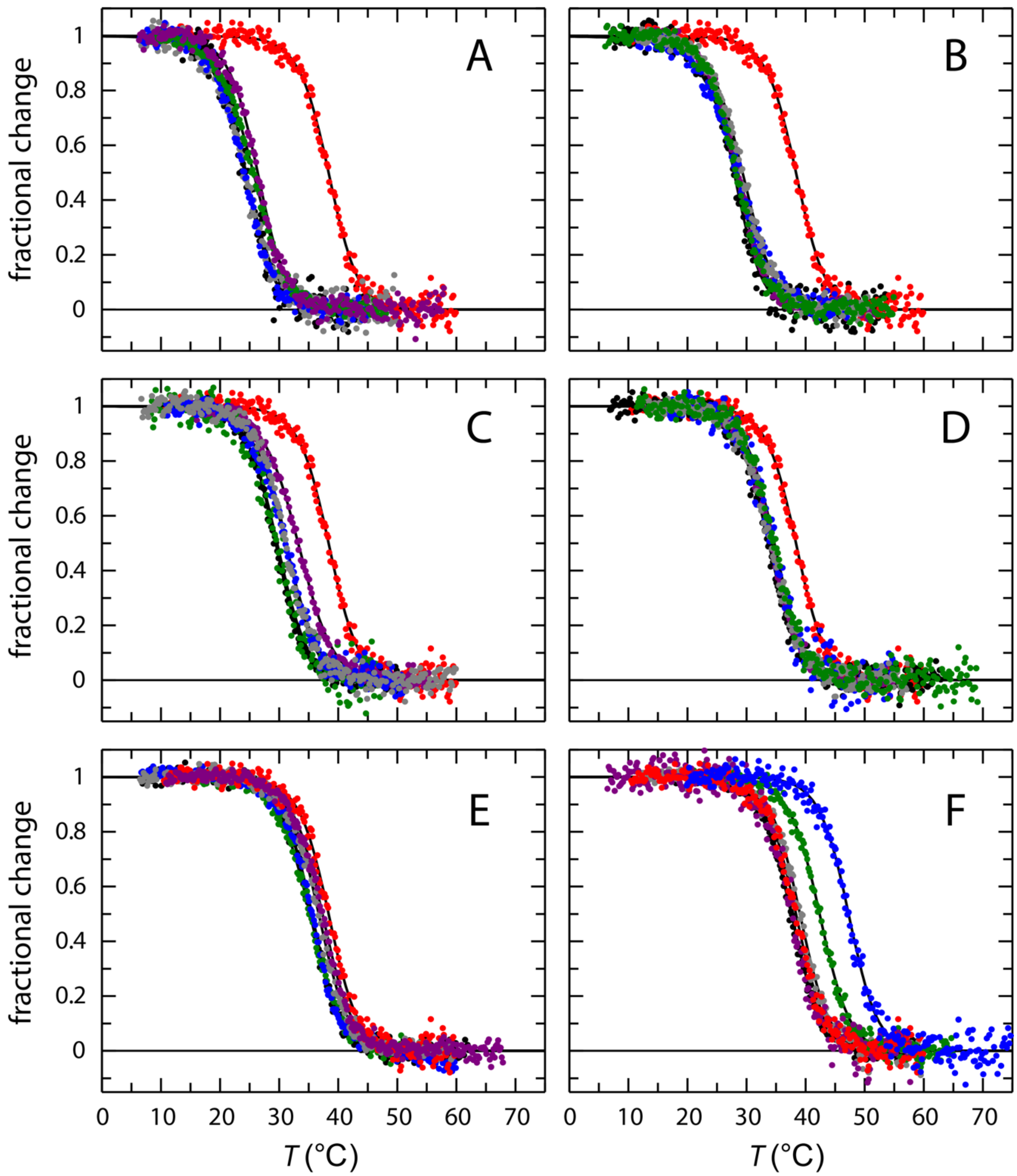

Supplementary Figure S2. Thermally induced unfolding transitions of N2' variants. The normalized unfolding transitions are shown as the fraction of native protein plotted against temperature. Analysis was performed using a two-state model (solid lines), with the baselines of the native protein fixed for the strongly destabilized variants N2'-L106A and N2'-V150A. The N2' variants are grouped in panels (a) to (f) by increasing stability: (**A**) (●) N2′-L106A; (●) N2′-M176A; (●) N2′-Y151A; (●) N2′-Y166A; (●) N2′-V150A; (**B**) (●) N2′-G146A; (●) N2′-I103A; (●) N2′-F185A; (●) N2′-T114A; (●) N2′-Y110A; (**C**) (●) N2′-G153A; (●) N2′-D187A; (●) N2′-Y203A; (●) N2′-F190A; (●) N2′-V155A; (D) (●) N2′-P121A; (●) N2′-N132A; (●) N2′-W181A; (●) N2′-P112A; (●) N2′-V200A; (**E**) (●) N2′-T169A; (●) N2′-H191A; (●) N2′-P123A; (●) N2′-Q116A; (●) N2′-N195A; (**F**)(●) N2′-P118A; (●) N2′-R142A; (●) N2′-L198P; (●) N2′-D178A; (●) N2′-Q145A. Thermal denaturation was monitored at 4 µM protein using CD spectroscopy at 222 nm in a quartz cuvette with a 1 cm path length. The experiment was conducted in 100 mM potassium phosphate buffer at pH 7.0. The heating rate was 60 K/h, with a bandwidth of 1.0 nm. Data points were recorded every 0.2 K.

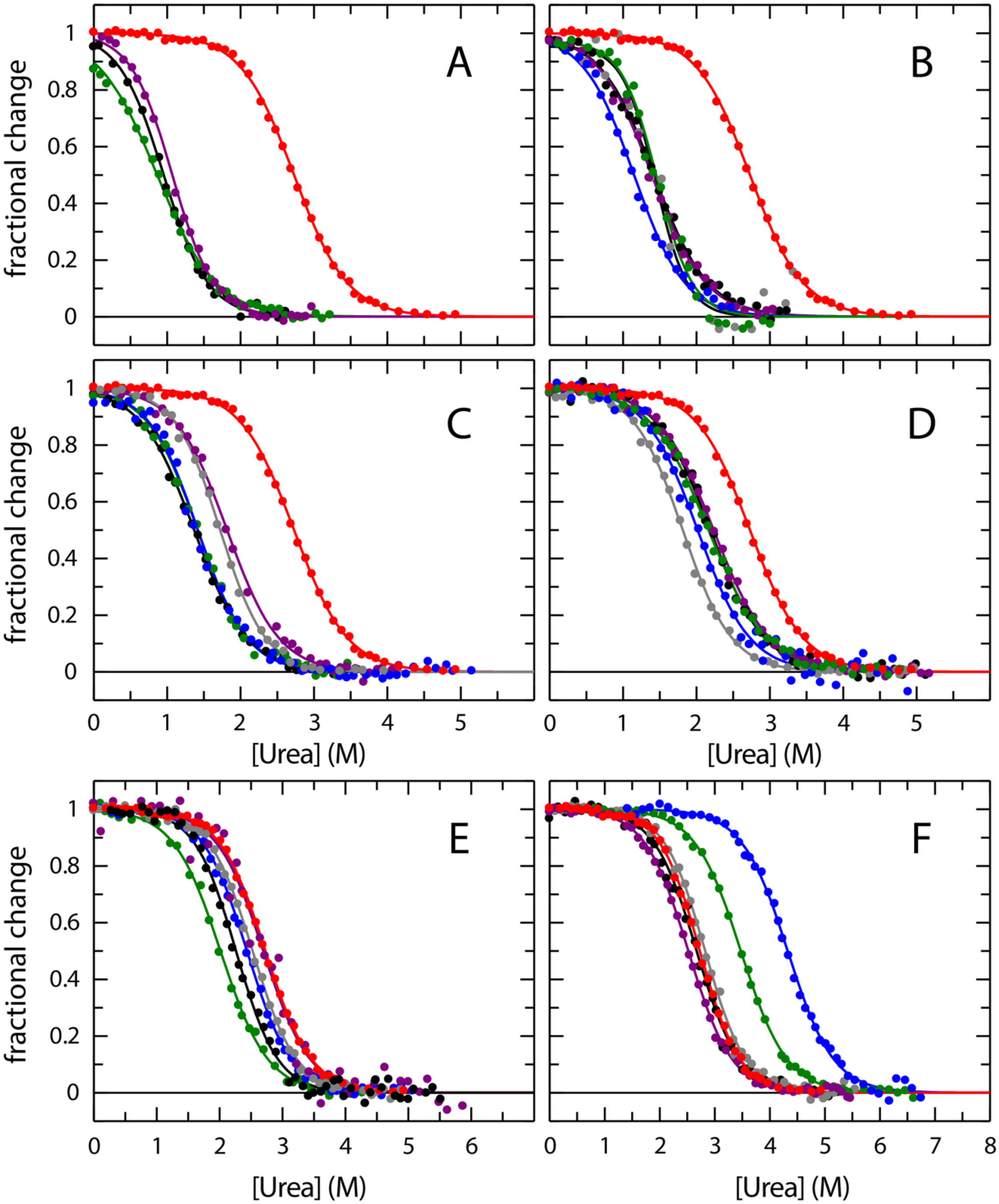

Supplementary Figure S3. Urea induced equilibrium unfolding transitions. The normalized unfolding transitions are shown as the fraction of native protein plotted against urea concentration. Fluorescence was measured at 340 nm after excitation at 280 nm at 15 °C (with the exception of N2'-W181A, measured at 305 nm). Analysis was performed using a two-state model (solid lines). (**A**) (●) N2′-L106A; (●) N2′-M176A; (●) N2′-Y151A; (●) N2′-Y166A; (●) N2′-V150A; (**B**) (●) N2′-G146A; (●) N2′-I103A; (●) N2′-F185A; (●) N2′-T114A; (●) N2′-Y110A; (**C**) (●) N2′-G153A; (●) N2′-D187A; (●) N2′-Y203A; (●) N2′-F190A; (●) N2′-V155A; (D) (●) N2′-P121A; (●) N2′-N132A; (●) N2′-W181A; (●) N2′-P112A; (●) N2′-V200A; (**E**) (●) N2′-T169A; (●) N2′-H191A; (●) N2′-P123A; (●) N2′-Q116A; (●) N2′-N195A; (**F**)(●) N2′-P118A; (●) N2′-R142A; (●) N2′-L198P; (●) N2′-D178A; (●) N2′-Q145A. The protein concentration was 0.5 μM or 1.0 μM (see Section 2.2.6.5) in 100 mM potassium phosphate buffer at pH 7.0. The excitation bandwidth was 3 nm, and the emission bandwidth was 5 nm.

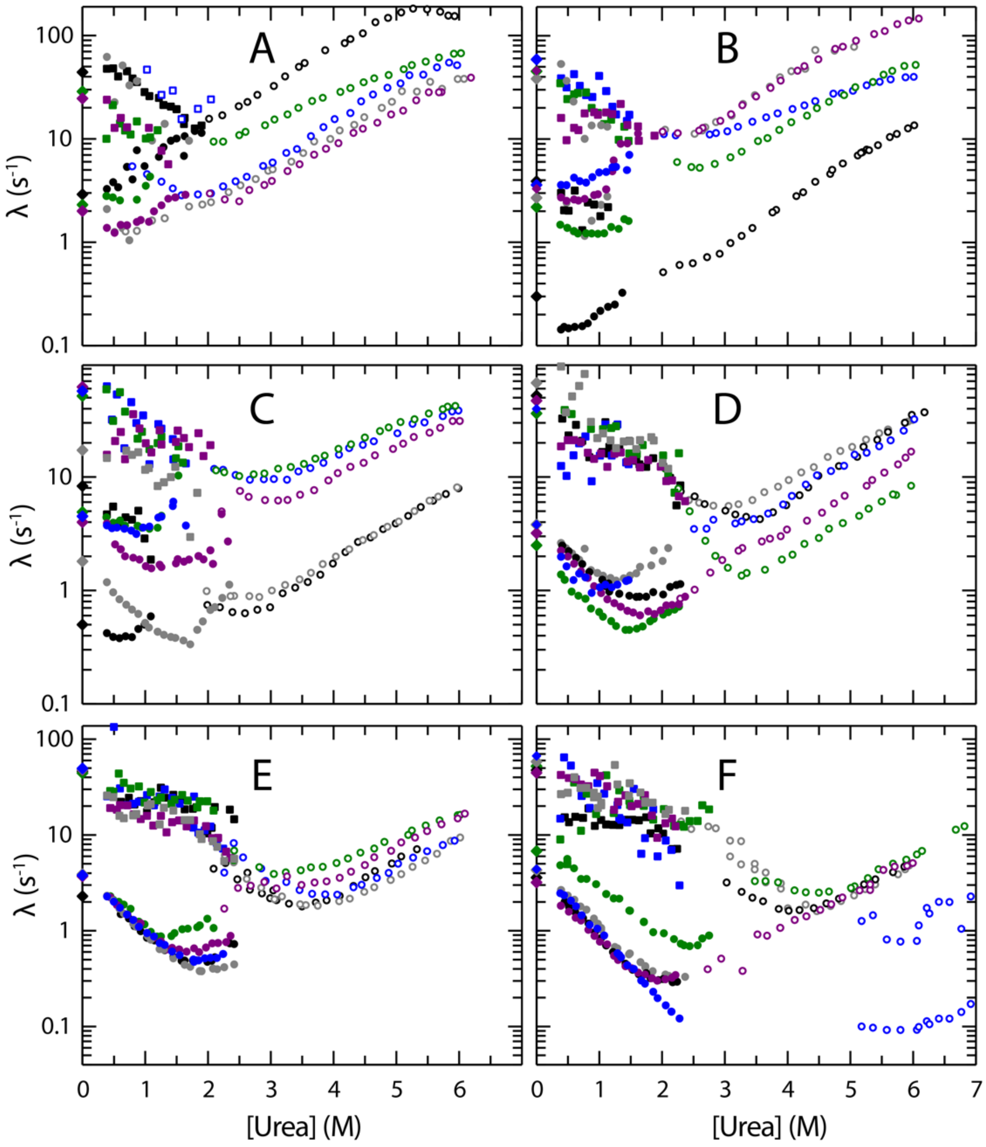

Supplementary Figure S4. Folding kinetics without chevron plot analysis. The folding kinetics were measured after stopped-flow single mixing by the changes in fluorescence above 320 nm (excitation at 280 nm). Plot of the apparent rates (λ in s^{-1}) for all N2' variants as a function of urea concentration (in M) (chevron plot). The fast (filled squares) and slow (filled circles) folding rates of the *cis*- and *trans*-conformers, respectively, are derived from the biexponential refolding kinetics and converge into the rates from the monoexponential unfolding kinetics (open circles). The N2' variants are grouped in panels (a) to (f) by increasing thermodynamic stability. (**A**) (●) N2′-L106A; (●) N2′-M176A; (●) N2′-Y151A; (●) N2′-Y166A; (●) N2′-V150A; (**B**) (●) N2′-G146A; (●) N2′-I103A; (●) N2′-F185A; (●) N2′-T114A; (●) N2′-Y110A; (**C**) (●) N2′-G153A; (●) N2′-D187A; (●) N2′-Y203A; (●) N2′-F190A; (●) N2′-V155A; (D) (●) N2′-P121A; (●) N2′-N132A; (●) N2′-W181A; (●) N2′-P112A; (●) N2′-V200A; (**E**) (●) N2′-T169A; (●) N2′-H191A; (●) N2′-P123A; (●) N2′-Q116A; (●) N2′-N195A; (**F**)(●) N2′-P118A; (●) N2′-R142A; (●) N2′-L198P; (●) N2′-D178A; (●) N2′-Q145A. Additionally, the rates of the N2' variants from the double-mixing experiments are shown in their respective colors (filled diamond). The kinetics of refolding and unfolding experiments were monitored after stopped-flow mixing (eleven-fold dilution) by measuring the fluorescence changes above 320 nm (or 300 nm for N2'-W181A) after excitation at 280 nm at 15 °C. The protein concentration in the measurement cell was 0.5 μM (or 1.5 μM for N2'-W181A) in 100 mM potassium phosphate buffer, pH 7.0, with the corresponding urea concentration.

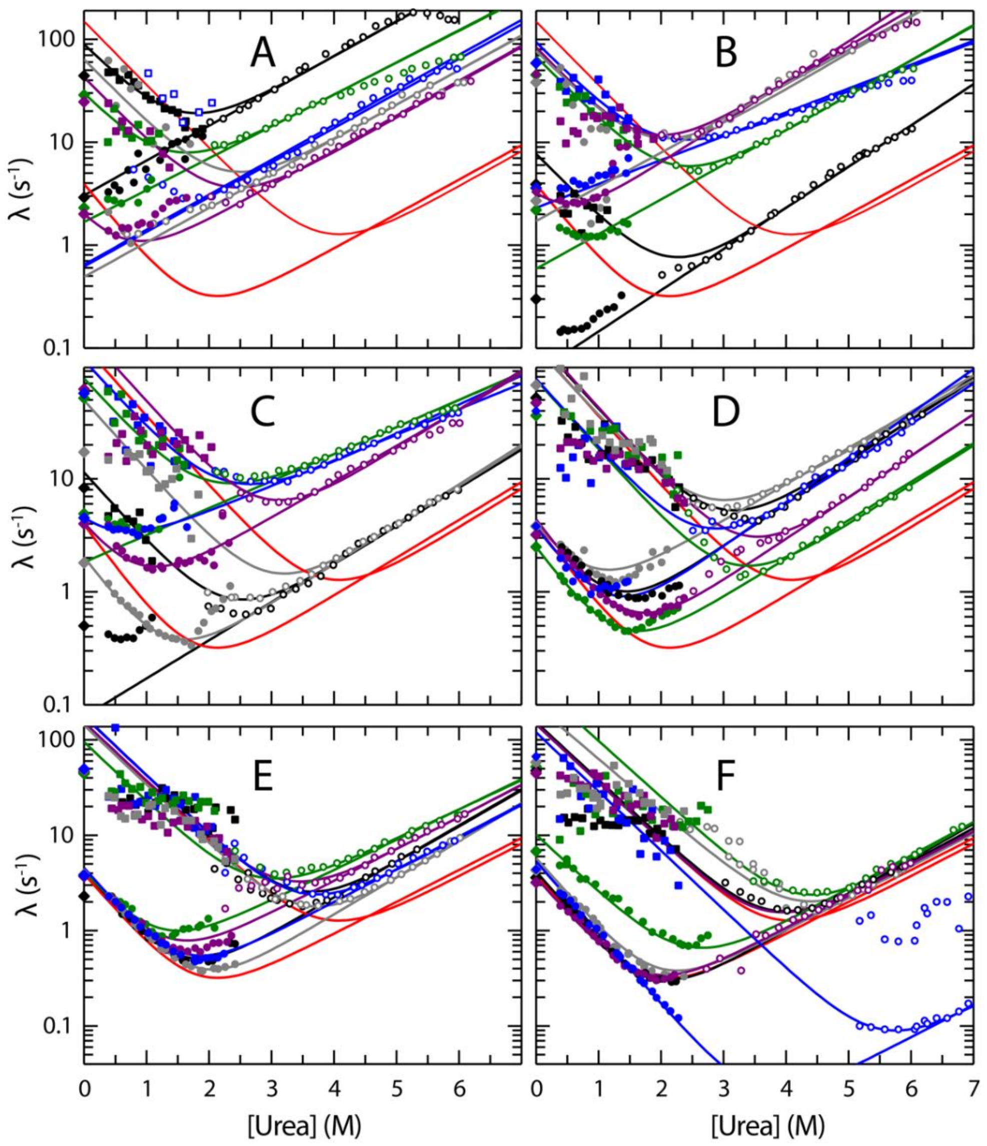

Supplementary Figure S5. The folding kinetics were measured after stopped-flow single mixing by the changes in fluorescence above 320 nm (excitation at 280 nm). Plot of the apparent rates (λ in s^{-1}) for all N2' variants as a function of urea concentration (in M) (chevron plot). The fast (filled squares) and slow (filled circles) folding rates of the *cis*- and *trans*-conformers, respectively, are derived from the biexponential refolding kinetics and converge into the rates from the monoexponential unfolding kinetics (open circles). The N2' variants are grouped in panels (a) to (f) by increasing thermodynamic stability. (**A**) (●) N2′-L106A; (●) N2′-M176A; (●) N2′-Y151A; (●) N2′-Y166A; (●) N2′-V150A; (**B**) (●) N2′-G146A; (●) N2′-I103A; (●) N2′-F185A; (●) N2′-T114A; (●) N2′-Y110A; (**C**) (●) N2′-G153A; (●) N2′-D187A; (●) N2′-Y203A; (●) N2′-F190A; (●) N2′-V155A; (D) (●) N2′-P121A; (●) N2′-N132A; (●) N2′-W181A; (●) N2′-P112A; (●) N2′-V200A; (**E**) (●) N2′-T169A; (●) N2′-H191A; (●) N2′-P123A; (●) N2′-Q116A; (●) N2′-N195A; (**F**)(●) N2′-P118A; (●) N2′-R142A; (●) N2′-L198P; (●) N2′-D178A; (●) N2′-Q145A. Additionally, the rates of the N2' variants from the double-mixing experiments are shown in their respective colors (filled diamond). The kinetics of refolding and unfolding experiments were monitored after stopped-flow mixing (eleven-fold dilution) by measuring the fluorescence changes above 320 nm (or 300 nm for N2'-W181A) after excitation at 280 nm at 15 °C. The protein concentration in the measurement cell was 0.5 μM (or 1.5 μM for N2'-W181A) in 100 mM potassium phosphate buffer, pH 7.0, with the corresponding urea concentration. The chevron analysis was performed using a two-state model (solid lines in the corresponding color of each N2' variant), and the results are summarized in Supplementary Table 2. For comparison, the respective fits for N2' wild-type (red line, —) are shown [1].

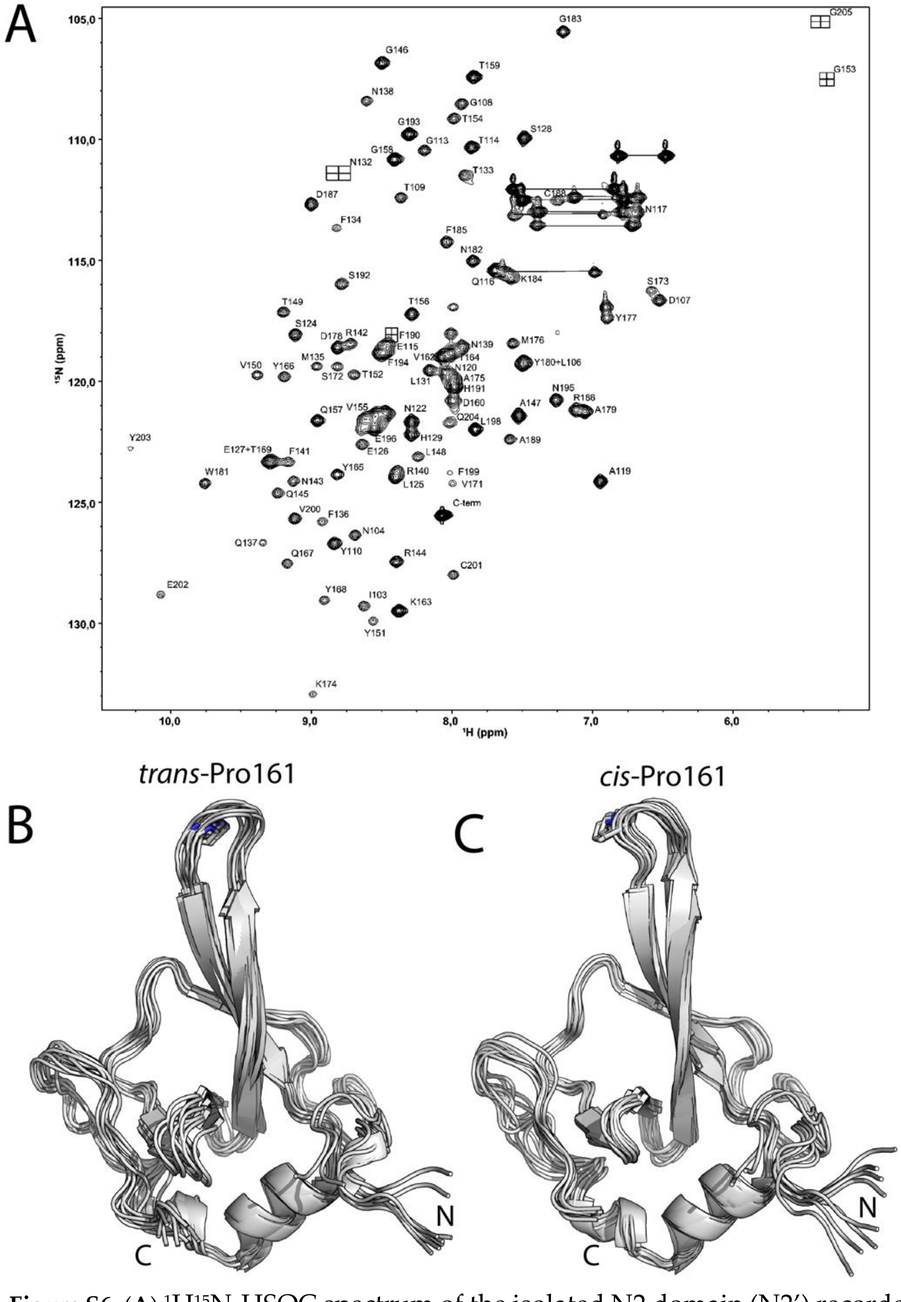

Supplementary Figure S6. (**A**) $^{1}H^{15}N$-HSQC spectrum of the isolated N2 domain (N2’) recorded at 15°C, pH7. Assigned amide cross signals are labeled. Signals with two low intensities at this contour level are indicated by boxes. Side chain NH_2 groups are connected by lines. Superposition of the tem lowest-energy models for *trans*-Pro161 (**B**) and *cis*-Pro161 (**C**). The N and C-terminus are indicated.

References

1. Jakob, R.P.; Schmid, F.X. Energetic coupling between native-state prolyl isomerization and conformational protein folding. *J Mol Biol* **2008**, *377*, 1560-1575, doi:10.1016/j.jmb.2008.02.010.
